# Exosomal long non-coding RNAs: novel molecules in gastrointestinal cancers’ progression and diagnosis

**DOI:** 10.3389/fonc.2022.1014949

**Published:** 2022-12-14

**Authors:** Mohammad Roshani, Ghazaleh Baniebrahimi, Mahboubeh Mousavi, Noushid Zare, Reza Sadeghi, Reza Salarinia, Amirhossein Sheida, Danial Molavizadeh, Sara Sadeghi, Farzaneh Moammer, Mohammad Reza Zolfaghari, Hamed Mirzaei

**Affiliations:** ^1^ Internal Medicine and Gastroenterology, Colorectal Research Center, Iran University of Medical Sciences, Tehran, Iran; ^2^ Department of Pediatric Dentistry, School of Dentistry, Tehran University of Medical Sciences, Tehran, Iran; ^3^ Department of Anatomy, Faculty of Medicine, Semnan University of Medical Sciences, Semnan, Iran; ^4^ Faculty of Pharmacy, Tehran University of Medical Science, Tehran, Iran; ^5^ School of Medicine, Tehran University of Medical Sciences, Tehran, Iran; ^6^ Department of Advanced Technologies, School of Medicine, North Khorasan University of Sciences, Bojnurd, Iran; ^7^ School of Medicine, Kashan University of Medical Sciences, Kashan, Iran; ^8^ Research Committee, Kashan University of Medical Sciences, Kashan, Iran; ^9^ Research Committee, School of Medicine, Guilan University of Medical Sciences, Rasht, Iran; ^10^ Department of Microbiology, Qom Branch, Islamic Azad University, Qom, Iran; ^11^ Center for Biochemistry and Nutrition in Metabolic Diseases, Institute for Basic Sciences, Kashan University of Medical Sciences, Kashan, Iran

**Keywords:** gastrointestinal cancer, non-coding RNAs, exosome, long non-coding RNAs, pathogenesis

## Abstract

Gastrointestinal (GI) cancers arise in the GI tract and accessory organs, including the mouth, esophagus, stomach, liver, biliary tract, pancreas, small intestine, large intestine, and rectum. GI cancers are a major cause of cancer-related morbidity and mortality worldwide. Exosomes act as mediators of cell-to-cell communication, with pleiotropic activity in the regulation of homeostasis, and can be markers for diseases. Non-coding RNAs (ncRNAs), such as long non-coding RNAs (lncRNAs), can be transported by exosomes derived from tumor cells or non-tumor cells. They can be taken by recipient cells to alter their function or remodel the tumor microenvironment. Moreover, due to their uniquely low immunogenicity and excellent stability, exosomes can be used as natural carriers for therapeutic ncRNAs *in vivo*. Exosomal lncRNAs have a crucial role in regulating several cancer processes, including angiogenesis, proliferation, drug resistance, metastasis, and immunomodulation. Exosomal lncRNA levels frequently alter according to the onset and progression of cancer. Exosomal lncRNAs can therefore be employed as biomarkers for the diagnosis and prognosis of cancer. Exosomal lncRNAs can also monitor the patient’s response to chemotherapy while also serving as potential targets for cancer treatment. Here, we discuss the role of exosomal lncRNAs in the biology and possible future treatment of GI cancer.

## Introduction

Gastrointestinal (GI) cancers, such as colorectal, esophageal, gastric, hepatocellular carcinoma, and pancreatic cancer, are among the most common malignancies diagnosed worldwide. Based on the GLOBOCAN cancer statistics, GI cancers account for 26% of newly diagnosed cases and 35% of cancer-related deaths globally (1, 2). Typically, chemotherapy, surgery, targeted therapy, and other approaches have been used to treat GI cancer. Patients with GI cancer who undergo early detection and therapy have a better prognosis than those who are diagnosed at an advanced stage (3, 4). Current diagnostic approaches mostly rely on invasive techniques that are difficult to apply for screening purposes, such as pathological biopsies or digestive tract endoscopy. The discovery of novel biomarkers, such as non-coding RNAs, is important for early diagnosis and more targeted therapy (4). The development of resistance to radiotherapy, chemotherapy, immunotherapy, or targeted therapy still poses a serious obstacle to effective cancer treatment, despite some recent advances. By deciphering crucial cellular signaling pathways, recent studies have demonstrated that ncRNAs also play a key role in the development of resistance to many cancer treatments (5).

Extracellular vesicles (EVs), also called exosomes, are formed when multivesicular bodies (MVBs) inside cells fuse with the plasma membrane. Their sizes range from 40 to 160 nm (average 100 nm) (6). It has been reported that B cells released antigen-presenting exosomes that triggered a T-cell response (7, 8). Moreover, exosomes could contain both messenger RNAs (mRNAs) and microRNAs (miRNAs) which could then be transported to other recipient cells in order to perform a specific function (9). Exosomes have been found to contain DNA, proteins, and RNAs, including non-coding RNAs (ncRNAs) and long-non coding RNAs (lncRNAs), which can allow cell-to-cell communication and affect signaling pathways (10).

The participation of exosomes in the development and progression of GI cancer has recently attracted more interest in both research and possible treatment. It has been hypothesized that cancer cells generate more exosomes compared with healthy cells (11). Exosomes are actively secreted by the source cancer cells and are crucial for cancer cell functioning. Exosomes can also be released by stromal cells, immune cells, or other cells in the cancer microenvironment. Exosome-mediated interactions between stromal cells, immune cells, and cancer cells have been implicated in the development of several GI cancers (11, 12).

**Figure 1:**
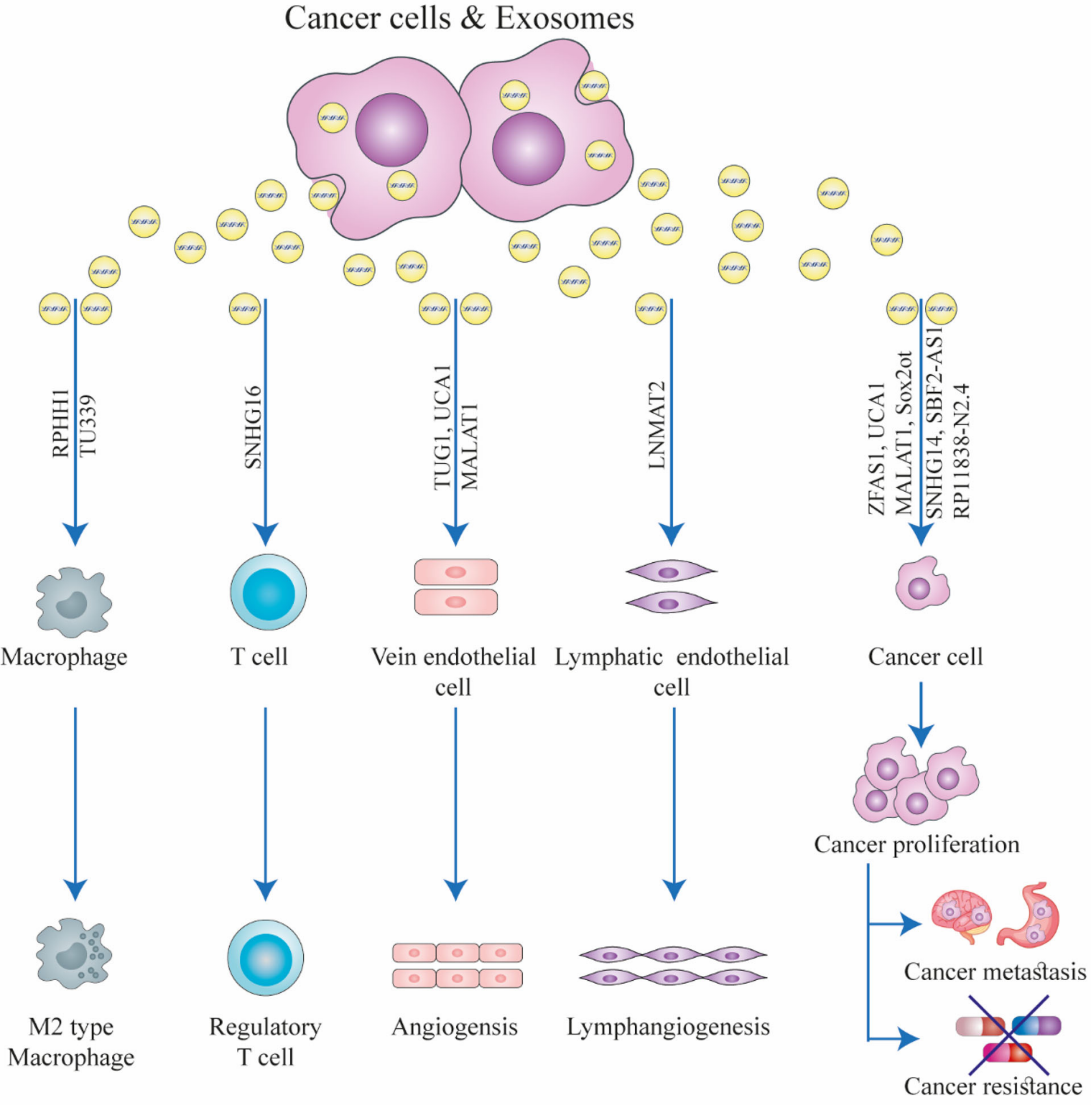
The function of exosomal long non-coding RNAs (lncRNAs) in the biology of cancer. Exosomes can facilitate interactions between stromal cells, immune cells, and cancer cells in the tumor microenvironment. Exosomal lncRNAs secreted from cancer cells can induce drug resistance, immunological regulation, angiogenesis, cancer growth, and metastasis in recipient cells. This figure was adapted from Zhang et al. (21).

lncRNAs show differential expression levels in cancer cells and normal tissues (13, 14). They can affect the function of target molecules, including miRNAs and proteins, and therefore, can regulate tumor aggressiveness and chemoresistance (15–17). The best known function of lncRNAs is their ability to act as competitive endogenous RNAs (ceRNA), by sponging their target miRNAs and thereby affecting the expression of the mRNA targets of the specific miRNAs (18). lncRNAs are essential regulatory elements in several biological processes, including RNA processing, transcriptional interference, chromatin remodeling, and protein degradation (19, 20). Many studies have concentrated on the significance of lncRNAs in cancer because they can regulate genetic and epigenetic alterations which are critical in carcinogenesis and cancer progression. lncRNAs are essential for the complete understanding of the biology of cancer (**Figure 1**). Abnormal expression of lncRNAs has been linked to tumor development, invasion, and overall patient survival by primarily affecting the epigenetic regulation of both oncogenes and tumor suppressor genes (21–25). Although some putative mechanistic roles have been clarified, the function of most lncRNAs still remains unclear (26). According to a recent study, lncRNAs may be regarded as complex molecular regulators or “fine-tuners” because of their ability to act in a tissue-/cell- specific manner (26–30). The role of lncRNAs in several GI cancers has been established. These lncRNAs may be divided into three classes based on their function, namely, tumor suppressors, tumor promoters (oncogenes), and dual-action tumor suppressors/promoters, and the latter precise function may be context- or tissue-dependent.

In this review, we will focus on exosome-enriched lncRNAs in the pathogenesis of GI cancers. We also briefly discuss the dysregulation of exosome-enriched lncRNA expression in GI cancer and summarize how exosomal lncRNAs can regulate cancer cell progression by acting as either tumor suppressors or as oncogenes.

## Exosome biogenesis

Exosomes are continuously produced from late endosomes generated by the invagination of the membrane of MVBs. This process subsequently leads to the formation of intraluminal vesicles (ILVs) inside the MVBs (31). During this process, the invaginated membranes engulf some specific proteins, while the same process occurs for some other cytosolic components which are then incorporated within the ILVs. A majority of ILVs are secreted into the extracellular space after their fusion to the cell membrane. The term “exosomes” refers to these secreted ILVs (32, 33).

Exosomes are a class of EVs with a diameter ranging from 30 to 100 nm (34). They are composed of a combination of lipids and proteins, which are derived from the endosomes from which they originated. The lipids are mainly composed of cholesterol, ceramide, and sphingomyelin (35–37). Canonical exosomes show a biconcave or cup-like shape, which is supposed to be due to a drying process taking place during their preparation for study. This is because they display a spheroidal shape in the aqueous environment under transmission electron microscopy (TEM) (38). Exosomes mainly show a density ranging from 1. 13 g/ml for B-cell-derived exosomes (39) to 1. 19 g/ml for epithelial cell-derived exosomes (40), as measured by sucrose gradient centrifugation. The proteins in the ESCRT (endosomal sorting complexes required for transport) play a role in the formation of exosomes. The ESCRT protein family is divided into four subgroups, i.e., ESCRT-0, ESCRT-I, ESCRT-II, and ESCRT-III (41). The detection and transfer of ubiquitinated proteins to specific domains located on the endosomal membrane by ubiquitin-binding subunits of ESCRT-0 has been found to initiate the ESCRT formation process. ESCRT-I then interacts with ESCRT-II forming a larger complex, which then unites with the ESCRT-III complex. The latter helps in the budding process. Eventually, the buds are cleaved from the membrane and form ILVs. Finally, the ESCRT-III complex is dissociated from the MVB membrane by the vacuolar protein sorting 4 (Vps4) AAA ATPase which provides the energy (42).

Some cargoes can be incorporated into MVBs without binding to ESCRT-0, ESCRT-I, or ESCRT-II protein complexes. Instead, ALG-2-interacting protein X (Alix), an ESCRT-interacting protein, binds to the CHMP4 subunit of the ESCRT-III complex and the pseudoautosomal region 1 (PAR1), a G-protein-coupled membrane receptor, and subsequently transfers PAR1 to MVBs without requiring ubiquitylation. ALIX also binds to syntenin causing syndecans to bind to CD63. The proteins ALIX, syntenin, syndecan, and CD63 are all found within MVBs and exosomes but do not require ubiquitination for their function (43, 44). ESCRT-independent pathways control the production of exosomes, which contain tetraspanin cargoes and require lipids for their activity.

Exosomes are rich in tetraspanins, proteins that possess four transmembrane domains each with a specific palmitoylation site. CD9, CD37, CD63, CD81, and CD82 (in addition to other tetraspanins) are considered to be specific biomarkers for exosomes, as they are concentrated on the exosome surface (9). Ceramides can trigger the separation of the lateral phase and the fusion of microdomains when studied in model membranes. Furthermore, the particular cone-shaped structure of exosomes is caused by ceramide, which spontaneously forms a negative curvature in the endosomal membrane, in order to promote domain-induced budding. This ceramide-based mechanism suggests an important role for exosomal lipids in the production of exosomes (45). Three glycosylphosphatidyl inositol (GPI)-anchored proteins (CD55, CD58, CD59), and the palmitoylated protein Lyn, are sorted into exosomes during the process of red blood cell maturation. Thus, proteins containing these lipids can selectively enter lipid rafts composed of cholesterol, sphingomyelin, and ceramides (46, 47). Higher levels of exosome secretion have been reported in human immunodeficiency virus type-1 (HIV-1)- infected cells, resulting in ESCRT-independent exosomal generation. Investigations have reported that two conventional markers of exosomes (tetraspanins CD63 and CD81) can be found within these exosomes, which have the same size as classical exosomal structures (46, 48). The ESCRT-0 complex is responsible for clustering the ubiquitinated cargoes (49).

## Exosomal long non-coding RNAs and different GI cancers

### Exosomal long non-coding RNAs and gastric cancer

Gastric cancer (GC) is the fifth most prevalent human malignancy and the third cause of cancer-related death according to the Global Cancer Observatory, CANCER TODAY (GLOBOCAN) 2018 statistics (50). Patients are frequently diagnosed with metastasis due to presenting at later stages (51). Most GC patients do not show any clinical symptoms at the early stages (52), while nausea and vomiting or upper GI symptoms are reported in some cases, but these are similar to peptic ulcer and so are not specific for GC (53). Therefore, most GC patients are at an advanced stage when the diagnosis is confirmed (54). Recent research has shown that exosome-derived lncRNAs are involved in the development, progression, and drug resistance of GC tumors. Exosomal lncRNA expression levels can also promote or inhibit the development of GC.

**Figure 2:**
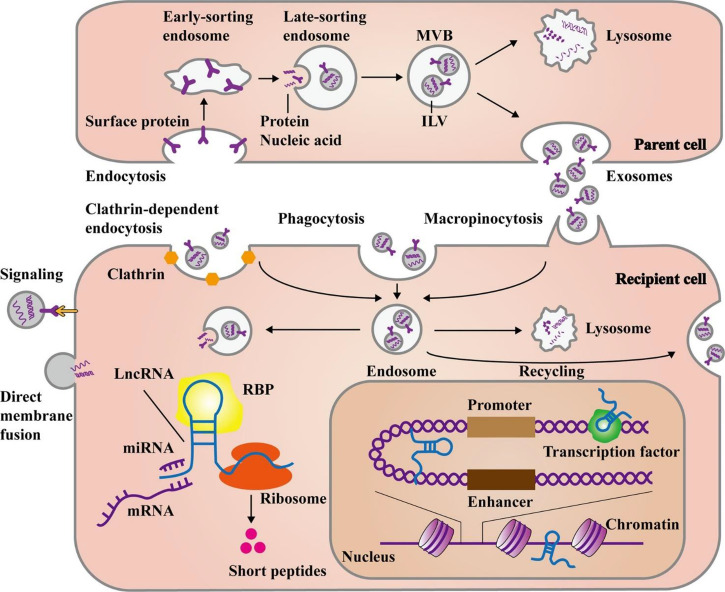
The main steps of biogenesis and release of exosomal lncRNAs. Exosome formation commences with the development of early and late-sorting endosomes by endocytosis of plasma membrane proteins. This process is followed by the formation of intraluminal vesicles (ILVs) from the late-sorting endosomes *via* the inward budding of their membrane which encapsulate macromolecules such as proteins, DNAs, and RNAs. Eventually, late-sorting endosomes develop into multivesicular bodies (MVBs) that secrete ILVs as exosomes. The MVBs can fuse with lysosomes for the degradation of their contents. Exosomes can either directly transmit their cargo to recipient cells or release it after fusion with the recipient cell plasma membrane. Moreover, exosomes may create endosomes by three main pathways, namely, endocytosis, macropinocytosis, or phagocytosis. In response to cellular requirements, the endosomes may release their exosomal contents, combine with lysosomes to be degraded, or fuse with cell membrane for recycling the exosomes. Then, the release of exosomal lncRNAs leads to the modulation of cell functions through several mechanisms. They can influence gene expression at post-transcriptional levels *via* targeting miRNAs, mRNAs, or proteins in the cytoplasm. Additionally, some lncRNAs have the potential for encoding short peptides. On the other hand, lncRNAs can play a role in the nucleus, by interacting with chromatins, transcription factors, or enhancer-like RNAs. This figure was adapted from Ahmadpour et al. (57).

Additionally, the exosomal membrane structures protect the lncRNAs from being broken down by enzymes in the body, thereby enhancing the stability of exosomal lncRNAs. Exosomes have distinctive characteristics that may be used to identify them, and their contents can be utilized to determine the cells from whence they originated. Exosomal lncRNAs can therefore be used as therapeutic targets, as well as prognostic or diagnostic biomarkers. Controlling exosome biogenesis and exosomal lncRNA expression levels may be a potential strategy to prevent or eliminate GC (55, 56). **Figure 2** illustrates the main steps of exosomal lncRNA biogenesis and release.

The forkhead box protein M1 (FOXM1) is a transcription factor which is evolutionarily conserved and involved in regulating cancer development and progression in various human malignancies (58–60). FOXM1 is upregulated in most human tumors, such as GC, and plays a substantial role in regulating the proliferation, migration, and apoptosis of cancer cells (61, 62). FOXM1 has already been reported in several studies to be a crucial oncogene correlated with the occurrence of GC, where it causes the upregulation of FOXM1-related LncRNA 1 (FRLnc1), an FOXM1-related lncRNA. This suggests that FRLnc1 could act as an independent prognostic indicator for the prediction of survival in GC patients (58, 63). Furthermore, it has been discovered that GC patient serum exosomes showed higher FRLnc1 expression (64). Importantly, FRLnc1 upregulation was significantly associated with clinicopathological properties, including lymph node metastasis and advanced TNM stage. Also, cellular assessment revealed that FRLnc1 knockdown using RNA interference reduced the proliferation and migration of HGC-27 cells, while its overexpression enhanced both properties in MKN45 cells. Following cellular treatment with exosomes, the expression of FRLnc1 was increased in MKN45 cells, and consequently, the proliferation and migration showed a significant increase. In conclusion, GC cell-derived exosomes were found to be involved in promoting the growth and metastasis of malignant cells through transporting the lncRNA FRLnc1, suggesting that exosome-transported FRLnc1 could act as a potential biomarker with possible diagnostic and therapeutic applications in GC patients. The transport of FRLnc1 *via* cancer cell-derived exosomes suggests that this process could be a novel therapeutic target for GC patients (64).

It has been demonstrated that the phosphoinositide 3-kinase (PI3K)/protein kinase B (AKT) signaling pathway plays a critical role in the initiation and development of several cancers and could therefore be a therapeutic target in cancer treatment. The development and prognosis of GC have been associated with abnormalities in PI3K/AKT pathways, which play a significant role in the progression and development of GC (65). Some nc RNAs have been shown to control this pathway (66–68). For example, Wang and colleagues assessed the possible role and mechanism of mesenchymal stem cell (MSC)-derived exosomal lncRNA LINC01559 in GC development (69). *In-silico* data retrieved from online databases including The Cancer Genome Atlas (TCGA) and the Gene Expression Profiling Interactive Analysis (GEPIA) showed the presence of LINC01559 in GC tissues. LINC01559 also demonstrated lower expression in GC cells compared with MSCs. Silencing studies showed that LINC01559 knockdown significantly decreased the proliferation, migration, and stemness of GC cells. The authors also found that LINC01559 was transported from MSCs to GC cells *via* exosomes, and their involvement was confirmed using immunofluorescence staining and electron microscopy. Mechanistically, LINC01559 was found to sponge miR-1343-3p, thereby upregulating *PGK1* (phosphoglycerate kinase 1) and eventually activating the PI3K/AKT pathway. Furthermore, LINC01559 recruited EZH2 [already well known to contribute to the development of human cancer (64, 65)] to the promoter of phosphatase and tensin homolog (*PTEN*) and induced its methylation, which eventually resulted in its repression. Therefore, LINC01559 enhanced GC progression by targeting both PGK1 and PTEN and, consequently, triggered the PI3K/AKT pathway. Overall, this study revealed that LINC01559 promoted GC progression through upregulation of PGK1 and downregulation of PTEN to activate the PI3K/AKT pathway, suggesting that LINC01559 could be a therapeutic target in GC (69).

The human stomach mucosa possesses a hypoxic environment, and GC tissues show even more severe hypoxia (70). The tissue architecture of GC is exceedingly heterogeneous, and immune cells, blood vessels, and fibroblasts interact intricately with cancer cells. The vessels and fibroblast cells of the tumor microenvironment affect O_2_ diffusion and perfusion and, consequently, result in hypoxia. Hypoxia is caused by a functional and structural abnormality of the tumor vasculature or disturbances in the regular geometry of the blood vessels (71). Hypoxia is one of the main properties of solid tumors linked to tumor growth, invasion, and metastasis (71). Recently, the molecular mechanism of tumor invasion in GC cells in a hypoxic environment was investigated (72). To simulate hypoxic conditions, GC cells were cultured in a medium treated with 1% O_2_, while normoxia conditions were created using 20% O_2_. The normoxic-cultured GC cells (NGCs) were co-cultured with medium from the hypoxia-cultured GC cells (HGCs). Cell scraping and Transwell tests were employed for the evaluation of the invasion and migration of GC cells. GC-derived exosomes were extracted using ultracentrifugation. The size distribution and exosome amounts were evaluated using electron microscopy and Western blot analysis. The prostate cancer gene expression marker 1 (PCGEM1) lncRNA was found to be highly expressed in HGC-derived exosomes. These exosomes increased the invasion and migration of NGCs. Mechanistically, PCGEM1 positively targeted the zinc finger transcription factor SNAI1, maintaining its stability and suppressing its degradation to induce the epithelial–mesenchymal transition (EMT) of GC cells. Taken together, PCGEM1 was upregulated in GC cells, partially incorporated into exosomes, to promote the migration and invasion of recipient GC cells. The authors suggested that exosomal PCGEM1 could act as a “scaffold” and, in combination with SNAI1, promote invasion and metastasis in GC (72).

**Figure 3:**
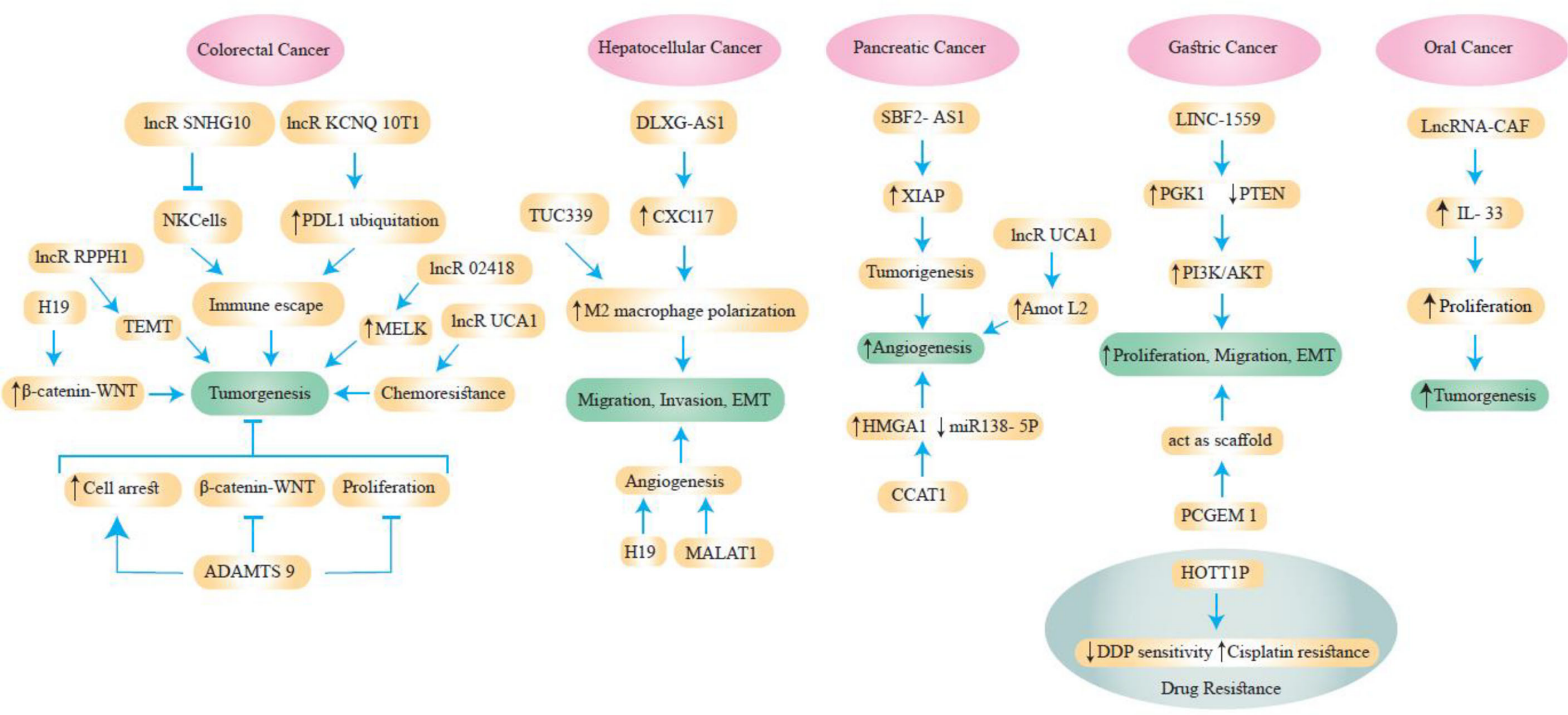
Some lncRNAs are involved in the pathogenesis of GI cancers.

Exosomal lncRNAs have a role in GC chemoresistance, similar to other ncRNAs (14, 73). According to Wang et al., downregulation of HOXA transcript at the distal tip (HOTTIP) increases cisplatin (DDP) sensitivity, whereas increased HOTTIP levels were seen in GC cells resistant to DDP (74). HOTTIP can activate DDP resistance by delivering exosomes from DDP-resistant GC cells to neighboring susceptible cells. Additionally, HOTTIP functions as a ceRNA, sponging miR-218 to activate HMGA1 in GC cells and increase resistance to cisplatin. Additionally, elevated serum expression of HOTTIP has been linked to DDP treatment side effects in GC patients (74). **Figure 3** and **Table 1** shows some lncRNAs that are involved in GI cancer pathogenesis.

**Table 1:** Summary of the involvement of lncRNAs in different GI cancers.

Cancers	lncRNAs	Expression in GI	Model	Cell line	Sample (type/number)	Ref
Gastric	H19	Upregul ation	Human	–	Serum/81	(75)
Gastric	PCGEM1	Up regulation	*In vitro*	AGS, MKN45	–	(72)
Gastric	Pcsk2-2:1	Downregul ation	Human	–	Serum/63	(76)
Gastric	GNAQ-6:1	Down regulation	Human	–	Serum/43	(77)
Gastric	CEBPA-AS1	Upregul ation	*In vitro*, human	GES-1, SGC-7901, BGC-823	Plasma/281, tissue/40	(78)
Gastric	MIAT	Upregul ation	Human	–	Serum/109	(79)
Gastric	HCG18	Upregul ation	*In vitro*	–	–	(80)
Gastric	HEIH	Upregul ation	*In vitro*, human	AGS, HGC-27, GES-1	Tissue/21	(81)
Gastric	HOTTIP	Upregul ation	Human	–	Serum/126	(82)
Gastric	LINC01559	Upregul ation	*In vitro*, *in vivo*, human	MKN74, NCI-N87, MKN-45, HGC-27, AGS	Tissue/80	(69)
Gastric	SPRY4-IT1	Upregul ation	*In vitro*, *in vivo*, human	GES-1, MKN28, SGC7901, BGC823	Tissue, serum/68	(83)
Gastric	ZFAS1	Upregul ation	*In vitro*, human	BGC-823, MGC-803, SGC-7901, MKN-28, GES-1	Tissue, serum/94	(84)
Gastric	GC1	Upregul ation	*In vitro*, human	MGC-803, SGC-7901, MKN-28, MKN-45, AGS, BGC-823, HGC-27, KATO III, HS-746T, SNU-5, GES-1	Tissue, serum/826	(85)
Gastric	lnc-SLC2A12-10:1	Upregul ation	*In vitro*, human	MGC803, BGC823, SGC7901, AGS, GES-1	Plasma/60, tissue/20	(86)
Gastric	UEGC1	Upregul ation	*In vitro*, human	AGS, KATO III, NCI-N87, Hs 746 T	Plasma/10	(87)
Gastric	FRLnc1	Upregul ation	*In vitro*, human	AGS, SNU-1, HS-746T, KATO III, NCI-N87, HGC-27, MKN45, GES-1	Tissue/60, blood/68	(64)
Gastric	LINC00152	Upregul ation	–	–	Plasma/79	(88)
Gastric	SND1−IT1	Upregul ation	*In vitro*, *in vivo*	GES-1	–	(89)
Gastric	X26nt	Upregul ation	*In vitro*, *in vivo*, human	BGC-823, MGC-803, MKN-45, GES-1	Tissue, serum/16	(90)
Gastric	NR038975	Upregul ation	*In vitro*, *in vivo*, human	AGS, MGC-803, BGC-823	Tissue/84	(91)
Gastric	RP11−323N12.5	Upregul ation	*In vitro*, *in vivo*, human	MKN45, MGC-803	Tissue/67	(92)
Pancreatic	NONHSAT105177	Downregul ation	*In vitro*, *in vivo*, human	HPDE, PDAC, SW1990, Capan1, PATU8988, HS766T, BXPC3, Panc1	Tissue/–	(93)
Pancreatic	ENST00000560647	Upregul ation	*In vitro*	PANC-1	–	(94)
Pancreatic	HULC	Upregul ation	*In vitro*, human	hTERT-HPNE, Panc-1, MiaPaCa-2, BxPC-3	Serum/42	(95)
Pancreatic	SBF2-AS1	Upregul ation	*In vitro*, *in vivo*	PANC-1, BxPC-3, SW1990, Capan-2, THP-1	–	(96)
Pancreatic	Sox2ot	Upregul ation	*In vitro*, *in vivo*, human	BxPC-3, Capan-1, Hs 766 T	Blood/61	(97)
Pancreatic	UCA1	Upregul ation	*In vitro*, *in vivo*, human	PANC-1, MIA PaCa-2, BxPC-3, Aspc-1, Sw1990, HEK293T	Serum/46	(98)
Pancreatic	CCAT1	Upregul ation	*In vitro*, *in vivo*, human	HPDE6-C7, PANC-1, BxPC-3, MIA PaCa-2, Capan-2	Tissue, serum/93	(99)
Pancreatic	MALAT1, CRNDE	Upregul ation	Human	–	Serum/–	(100)
Pancreatic	SNHG11	Upregul ation	*In vitro*, *in vivo*	HPDE6-C7, PANC-1, AsPC1, SW1990, HS766T	–	(101)
Pancreatic	linc-ROR	Upregul ation	*In vitro*, *in vivo*, human	CCC-HPE-2, PANC-1, AsPC-1, MIA-PACA-2, CFPAC-1, BxPC-3, HEK293T, 3T3-L1	Serum/48	(102)
Pancreatic	LINC01133	Upregul ation	*In vitro*, *in vivo*, human	CFPAC-1, AsPC-1, Panc-1, SW1990, HPDE	Tissue/32	(103)
Pancreatic	LINC00623	Upregul ation	*In vitro*, *in vivo*, human	AsPC-1, BxPC-3, CFPAC-1, MIAPaCa-2, PANC-1, HPNE, HEK-293T	Tissue/133, serum/73	(104)
Colorectal	HOTTIP	Downregul ation	Human	–	Serum/100	(105)
Colorectal	LNCV6_116109, LNCV6_98390, LNCV6_38772, LNCV_108266, LNCV6_84003, LNCV6_98602	Upregul ation	Human	–	Plasma/50	(106)
Colorectal	H19	Upregul ation	*In vitro*, *in vivo*, human	HCT116, SW480	Tissue/10	(107)
Colorectal	GAS5	Upregul ation	Human	–	Tissue, serum/158	(108)
Colorectal	CRNDE, H19, UCA1, HOTAIR	Upregul ation	Bioinformatics	HCT116, HT29, LoVo	–	(109)
Colorectal	CRNDE-h	Upregul ation	*In vitro*, *in vivo*, human	HCT116, SW480 SW620, HT-29, LoVo, FHC	Tissue/50, serum/148	(110)
Colorectal	CRNDE-p	Upregul ation	*In vitro*, *in vivo*, human	NCM460, HT-29, SW480, HCT-116, SW620, LoVo, SW48, DLD-1, Caco2, HT-15	Serum/411	(111)
Colorectal	MALAT1	Upregul ation	*In vitro*, *in vivo*, human	LoVo, HCT-8, SW620, SW480	Tissue/45	(112)
Colorectal	KCNQ1OT1	Upregul ation	*In vitro*, *in vivo*, human	FHC, HEK-293T, SW480, SW1463, HT-29, CT26	Tissue/20	(113)
Colorectal	SNHG10	Upregul ation	*In vitro*, *in vivo*, human	SW480, NK92-MI	Tissue/30	(114)
Colorectal	HOTTIP	Upregul ation	*In vitro*, *in vivo*, human	HCT116, SW620, LoVo, HT29, SW480, SW1116, Caco2	Tissue, blood/95	(115)
Colorectal	CCAT2	Upregul ation	Human	–	Tissue, blood/75	(116)
Colorectal	NNT−AS1	Upregul ation	*In vitro*, human	HCnEpC, LoVo, RKO, SW48, HCT116	Tissue, blood/40	(117)
Colorectal	LINC02418	Upregul ation	*In vitro*, human	DLD-1, SW480, HT29, HCT116, SW1116, LOVO, FHC, HEK293T	Tissue/60, blood/155	(118)
Colorectal	LINC00659	Upregul ation	*In vitro*	LOVO, SW48	–	(119)
Colorectal	UCA1	Upregul ation	*In vitro*, *in vivo*, human	HCT116, DLD1, SW480, RKO, HT-29, HCoEpiC, 293T	Tissue, blood/68	(120)
Colorectal	RPPH1	Upregul ation	*In vitro*, *in vivo*, human	HCT8, SW620, HT29, 293T	Tissue/61	(121)
Colorectal	APC1	Downregul ation	*In vitro*, *in vivo*, human	HCT116, DLD-1, SW480, LOVO, SW1116	Tissue/110	(122)
Colorectal	CCAL	Upregul ation	*In vitro*, *in vivo*, human	SW480, HCT116, HEK293T	Tissues/30	(196)
Colorectal	ADAMTS9-AS1	Downregul ation	*In vitro*, *in vivo*, human	DLD‐1, SW480, HT29, HCT116, SW1116, LOVO	Tissue/109, serum/130	(123)
Colorectal	91H	Upregul ation	*In vitro*, human	HCT-8, HCT-116, FHC	Serum/232	(124)
Colorectal	FOXD2-AS1, NRIR, XLOC_009459	Upregul ation	Human	–	Serum/203	(125)
Colorectal	LINC00152	Downregul ation	*In vitro*, human	SW480-7	Serum/18	(126)
Colorectal	lnc-HOXB8-1:2	Upregul ation	*In vitro*, human	LoVo, 293 T, THP-1	Tissue/105	(127)
Colorectal	LINC01315	Upregul ation	*In vitro*	SW480, HCT116	–	(128)
Colorectal	PCAT1	Upregul ation	*In vitro*, *in vivo*	HUVEC, NCM460, HCT116, SW480, T84	–	(129)
Colorectal	WEE2-AS1	Upregul ation	*In vitro*, *in vivo*, human	HCT 116, HT-29, HEK293T	Tissue, plasma/50	(130)
Colorectal	PVT1	Upregul ation	*In vitro*, *in vivo*, human	HCT116, LoVo	Tissue, serum/40	(131)
Colorectal	SPINT1-AS1	Upregul ation	Human	–	Tissue/150, serum/45	(132)
Esophageal	ZFAS1	Upregul ation	*In vitro*, *in vivo*, human	EC9706, Eca109, TE-13, TE-1, TTN	Tissue/136	(133)
Esophageal	PART1	Upregul ation	*In vitro*, *in vivo*, human	TE1, TE6, TE8, TTn, KYSE-450	Serum/79	(134)
Esophageal	POU3F3	Upregul ation	*In vitro*, human	KYSE450, TE12, Het-1a	Blood/78	(135)
Esophageal	NR_039819, NR_036133, NR_003353, ENST00000442416.1, ENST00000416100.1	Upregul ation	Human	–	Tissue/20, blood/317	(136)
Esophageal	AFAP1-AS1	Upregul ation	*In vitro*, *in vivo*	PBMC, KYSE410	–	(137)
Esophageal	UCA1	Downregul ation	*In vitro*, *in vivo*, human	EC18, Kyse140	Tissue/15, plasma/30	(138)
Esophageal	LINC01711	Upregul ation	*In vitro*, *in vivo*, human	ESC	Tissue/137	(139)
Esophageal	PCAT1	Upregul ation	*In vitro*, *in vivo*, human	KYSE30, KYSE70, KYSE140, KYSE150, KYSE180, KYSE410, KYSE450, KYSE510, COLO680N	Tissue, serum/39	(140)
Esophageal	FMR1-AS1	Upregul ation	Human	–	Tissue/394	(141)
Esophageal	RP5-1092A11.2	Upregul ation	*In vitro*, human	EC109, KYSE30, KYSE150	Tissue, plasma/6	(142)
Esophageal	FAM225A	Upregul ation	*In vitro*, human	ECA109, TE-1, KYSE150, KYSE-410, HET-1A, HUVEC	Tissue/30	(143)
Esophageal	RP11-465B22.8	Upregul ation	*In vitro*, *in vivo*, human	TE-1, KYSE-150, KYSE-510, HEEC, THP-1	Tissue/26	(144)
Oral	TIRY	Upregul ation	*In vitro*, human	TCA8113	Tissue/145	(145)
Oral	CAF	Upregul ation	*In vitro*, *in vivo*, human	HSC-3	Tissue/140	(146)
Oral	APCDD1L-AS1	Upregul ation	*In vitro*, human	SCC-4, HSC-3, TSCC1, SCC090, HN-4, NHOK	Tissue/40	(147)
Oral	LBX1-AS1	Upregul ation	*In vitro*, *in vivo*	SCC-4, CAL-27	–	(148)
Hepatocellular	FAL1	Upregul ation	*In vitro*, human	HepG2.2.15, LO2, Huh7, HepG2, SMMC-7721	Tissue, serum/30	(149)
Hepatocellular	TUC339	Upregul ation	*In vitro*	THP-1, U937, HL-7702	–	(150)
Hepatocellular	H19	Upregul ation	*In vitro*	HUVECs, Huh7, Sk-Hep	–	(151)
Hepatocellular	LUCAT1, CASC9	Upregul ation	*In vitro*, human	HepG2, Hep3B, SNU398, SNU449, SNU182, SNU475, PLC/PRF5, Huh-7	Tissue/60, serum/14	(152)
Hepatocellular	Linc-ROR	Upregul ation	*In vitro*	HepG2, PLC-PRF5	–	(153)
Hepatocellular	CTD-2116N20.1, AC012074.2, RP11-538D16.2, LINC00501, RP11-136I14.5	Upregul ation	Human	–	Tissue/364	(154)
Hepatocellular	ASMTL-AS1	Upregul ation	*In vitro*, *in vivo*, human	HepG2, Huh7, HCCLM3, SMMC7721, THLE-2	Tissue/70	(155)
Hepatocellular	CRNDE	Upregul ation	Human	–	Serum/166	(156)
Hepatocellular	SENP3-EIF4A1	Downregul ation	*In vitro*, *in vivo*, human	HL-7702, SMMC-7721, MHCC97L, HuH7, Hep3b	Tissue, plasma/50	(157)
Hepatocellular	LINC00161	Upregul ation	Human	–	Serum/20	(158)
Hepatocellular	linc-FAM138B	Downregul ation	*In vitro*, *in vivo*, human	SK-Hep-1, HepG2	Tissue/40	(159)
Hepatocellular	DLX6-AS1	Upregul ation	*In vitro*, *in vivo*, human	SMMC-7721, HepG2, HL-7702, THP-1	Tissue/76	(160)
Hepatocellular	MALAT1	Upregul ation	*In vitro*, *in vivo*, Human	HUVEC, Huh-7, Hep3B	Tissue/82	(161)
Hepatocellular	ENSG00000248932.1, ENST00000440688.1 ENST00000457302.2	Upregul ation	Human	–	Blood/200	(162)
Hepatocellular	ATB	Upregul ation	Human	–	Serum/79	(163)
Hepatocellular	HULC	Upregul ation	*In vitro*, human	HepG2, SMMC7721, LO2	Tissue, serum/30	(164)
Hepatocellular	MMPA	Upregul ation	*In vitro*, *in vivo*, human	Hep3B, BEL7404	Tissue/265	(165)
Hepatocellular	FAM72D-3	Upregul ation	*In vitro*, human	HepG2, Hep3B, SNU-423, PLC/PRF/5	Serum/45	(166)
Hepatocellular	EPC1-4	Downregul ation	*In vitro*, human	HepG2, Hep3B, SNU-423, PLC/PRF/5	Serum/45	(166)
Hepatocellular	ENSG00000258332.1, LINC00635	Upregul ation	Human	–	Serum/55	(167)
Hepatocellular	RN7SL1	Upregul ation	Human	–	Plasma/77	(168)
Hepatocellular	SNHG16	Upregul ation	*In vitro*, *in vivo*, human	Huh-7	Tissue/23, plasma/10	(169)
Hepatocellular	PCED1B-AS1	Upregul ation	*In vitro*, human	Huh-7, HepG2	Tissue/45	(170)
Hepatocellular	TUG1	Upregul ation	*In vitro*, *in vivo*, human	HepG2	Tissue/120	(171)
Hepatocellular	TUC339	Upregul ation	*In vitro*	Hep3B, HepG2, PLC/PRF/5	–	(172)
Hepatocellular	RP11-583F2.2	Upregul ation	Human	–	Serum/60	(173)

### Exosomal long non-coding RNAs and pancreatic cancer

Pancreatic cancer (PC) is among the leading causes of cancer-related death in developed countries and is classified into two major types, namely, adenocarcinoma (85% of diagnosed cases) and pancreatic endocrine tumors (<5%) (174). Tobacco, obesity, alcohol consumption, age, heredity, and chronic pancreatitis are the main risk factors for PC (175). Anorexia, asthenia, abdominal pain, and weight loss are the main clinical symptoms of the disease, but PC is not easily diagnosed due to a lack of specific symptoms (176). Additionally, little progress has been made in discovering preventative or therapeutic approaches for patients with PC, especially advanced PC (177). Given the role of ncRNAs in the development and progression of PC, pancreatic ductal adenocarcinoma (PDAC) has been reported to be associated with some exosomal lncRNAs (178).

Tumor-associated macrophages (TAMs), including M2 phenotype cells, are found to infiltrate solid tumors and help to induce proliferation, invasion, and angiogenesis (179, 180). M2-polarized TAMs are correlated with a poor prognosis in PC because of their role in lymphatic metastasis (181). Exosomes are also found in the bloodstream and tissue microenvironment, and it has recently been proposed that exosomes produced by M2 macrophages provide tumor cells with a regulatory transfer of proteins or signals to regulate their migration. For instance, the exosome- induced transfer of ApoE protein from TAMs to PC cells enhanced cell migration (48, 182). The mechanism through which M2 macrophages affect the proliferation, migration, and invasion of PC cells *via* the SBF2-AS1/miR-122-5p/XIAP axis (XIAP is an inhibitor of apoptosis proteins) has been explored (96). LPS plus IFN-γ and interleukin-4 treatment were used for transforming THP-1 cells into M1 and M2 macrophages. The PANC-1 PC cell line highly expressing lncRNA SET-binding factor 2 antisense RNA1 (SBF2-AS1) was selected as a tumor model for the extraction and identification of M2 macrophage-derived exosomes. SBF2-AS1 and XIAP expression levels, the SBF2-AS1 effects on the malignant phenotype of PC cells, and interactions between SBF2-AS1, miR-122-5p, and XIAP were evaluated. The effect of M2 macrophage exosomal SBF2-AS1 on the malignant phenotype of PANC-1 tumors was assessed *in vivo*. The results demonstrated that M2 macrophage-derived exosomes increased PC cell proliferation, migration, and invasion. SBF2-AS1 overexpression also showed the same effect on the progression of PC cells. Mechanistically, SBF2-AS1 repressed miR-122-5p expression and acted as a ceRNA to eventually upregulate XIAP expression. Additionally, M2 macrophage-derived exosomes containing SBF2-AS1 restrained the malignant phenotype of PC cells. Taken together, this study demonstrated that SBF2-AS1 incorporated into M2 macrophage-derived exosomes sponged miR-122-5p to upregulate XIAP, which acted as an inhibitor of PC progression (96).

**Figure 4:**
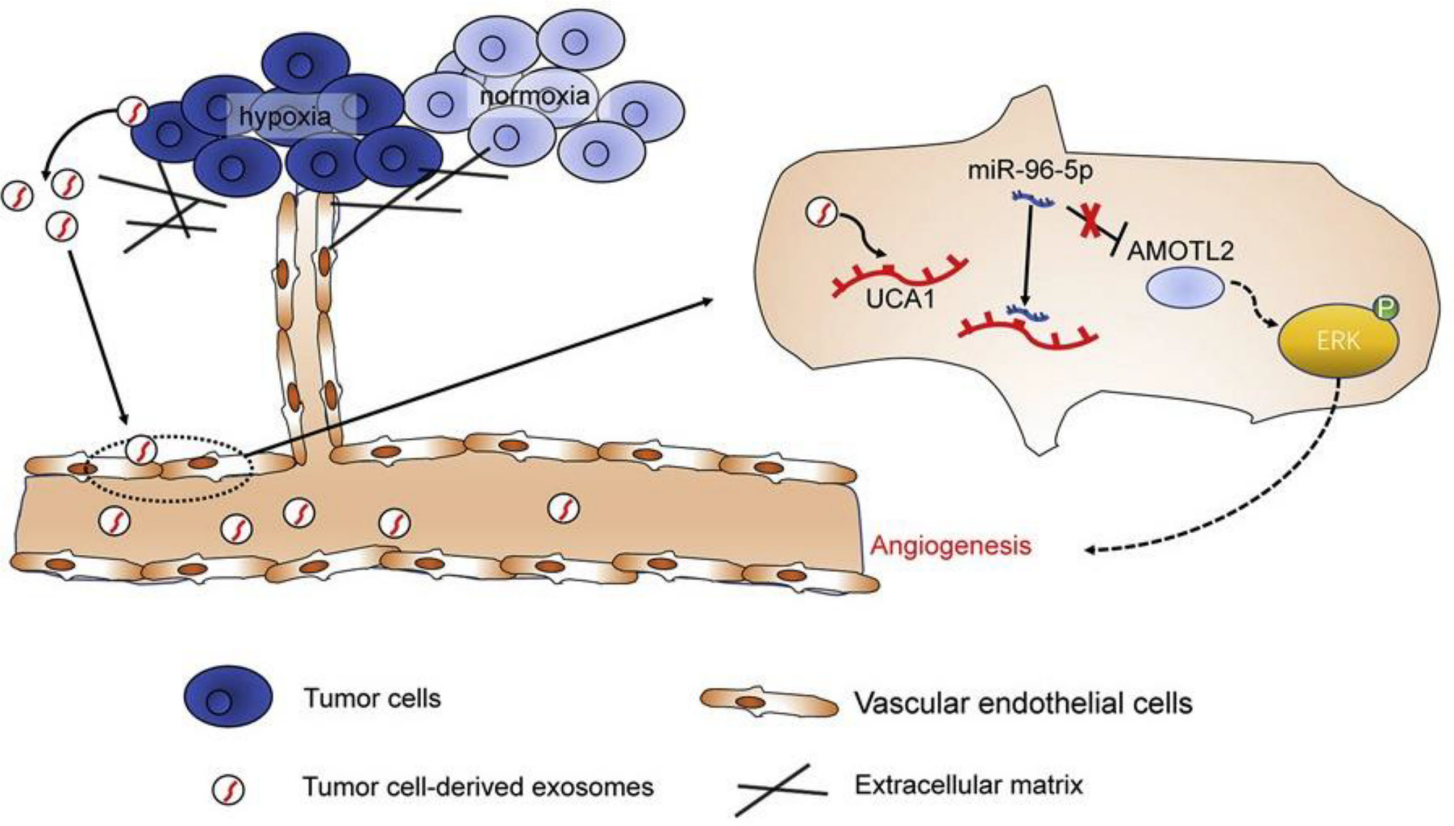
The lncRNA-UCA1 derived from exosomes of hypoxic cancer cells induces angiogenesis *via* the miR-96-5p/AMOTL2 axis in pancreatic cancer (PC). In summary, the hypoxic microenvironment, a crucial component of solid tumors, encourages the release of exosomes from tumor cells and increases tumor angiogenesis. Human umbilical vein endothelial cells (HUVECs) responded well to hypoxic exosomes produced by PC cells in terms of tube formation and cell migration. Exosomes derived from hypoxic PC cells were shown to have significant levels of the long non-coding RNA (lncRNA) UCA1, which could then be transferred to HUVECs *via* the exosomes. microRNA (miR)-96-5p was sponged by UCA1, which counteracted the suppressive effects of miR-96-5p on its target gene AMOTL2 expression. Hypoxic exosomal UCA1 might act as a potential target for PC therapy by promoting tumor development and angiogenesis through the miR-96-5p/AMOTL2/ERK1/2 axis. This figure was adapted from Guo et al. (85).

Angiomotin (AMOT) is an angiostatin-binding protein, also known as a motin (183). This family of AMOTs has three members: AMOT, AMOT-like 1 (AMOTL1), and AMOTL2 (183). AMOTL2 has been found to be involved in the regulation of vascular endothelial cell proliferation, polarity, and tube formation, which are due to positive regulation of the MAPK/ERK1/2 signaling pathway (184). Moreover, AMOTL2 has been correlated with the progression and occurrence of some human cancers (185–187). Guo and colleagues investigated the role of exosomes produced by hypoxic PC cells in the angiogenesis of PC tumors (98). They reported that PC cell-derived hypoxic exosomes promoted the migration and tube formation of human umbilical vein endothelial cells (HUVECs) (98). The lncRNA UCA1 was found to be highly expressed in these exosomes and could be transferred to HUVECs. Additionally, the UCA1 expression levels in exosomes isolated from serum samples of PC patients were higher than those in healthy individuals, while it was correlated with worse prognosis in PC patients. Furthermore, hypoxic exosomal UCA1 increased the proliferation of cancer cells, as well as angiogenesis and tumor growth in a mouse xenograft model. Mechanistically, UCA1 sponged miR-96-5p, to upregulate AMOTL2 expression. Overall, these findings demonstrated that hypoxic exosomal UCA1 may enhance angiogenesis and tumor growth in PC *via* the miR-96-5p/AMOTL2/ERK1/2 axis and so could be a novel therapeutic target for PC (**Figure 4**) (13, 98).

Another study investigated the role of exosomal lncRNA colon cancer-associated transcript-1 (CCAT1) isolated from PANC-1 cells, in PC tumorigenesis *via* its regulatory effect on the miR-138-5p/HMGA1 axis (99). Tissues retrieved from cancer patients and normal healthy tissues were used to compare CCAT1 and miR-138-5p expression levels. Plasmids were used to alter CCAT1 and/or miR-138-5p expression in PANC-1 cells. Isolated exosomes from PANC-1 cells were co-cultured with HUVECs, and proliferation and apoptosis in both cells were assessed. Also, the angiogenesis function of HUVECs was examined *in vitro* and *in vivo*. CCAT1, miR-138-5p, and HMGA1 expression levels were measured, as well as their interactions. The qRT-PCR results demonstrated that CCAT1 and HMGA1 were upregulated, while miR-138-5p was downregulated in PC cells and tissues. CCAT1 knockdown suppressed proliferation while inducing apoptosis in PANC-1 cells. PANC-1 cell-derived exosomal CCAT1 significantly reduced the proliferation and increased the apoptosis in HUVECs. Downregulation of exosomal CCAT1 suppressed the angiogenic function of HUVECs both in cell culture and xenograft mouse models, while CCAT1 upregulation reversed these effects and promoted angiogenesis. Mechanistically, CCAT1 upregulated HMGA1 through sponging miR-138-5p. Interestingly, miR-138-5p overexpression reversed the promoting effects of CCAT1 on the angiogenic ability of HUVECs *in vitro*. Collectively, this study suggested that PANC-1 cell-derived exosomal CCAT1 promoted the angiogenic function of HUVECs *via* the miR-138-5p/HMGA1 axis (99).

### Exosomal long non-coding RNAs and colorectal cancer

Colorectal cancer (CRC) is the third most common human malignancy and ranks as the fourth cause of cancer-related mortality around the globe (50, 188, 189). Although intensive investigations and some therapeutic advances have been made, still more than 50% of CRC patients do not survive, which is mainly due to late diagnosis and treatment (190). Therefore, early diagnosis could improve survival in CRC patients. The current diagnostic methods for CRC detection and screening, such as carcinoembryonic antigen (CEA) blood test and colonoscopy, are limited in their clinical application due to low diagnostic power, high costs, and invasive procedures producing discomfort (191, 192). Discovering the molecular mechanisms involved in the pathogenesis of CRC may help to develop novel biomarkers with improved potential for patient diagnosis (193). A number of lncRNAs are known to be involved in the underlying mechanisms responsible for the development and progression of CRC (194). Exosomal lncRNAs may also function as prognostic biomarkers in patients with CRC. For instance, a recent work has investigated the role of circulating exosomal lncRNAs as new CRC biomarkers and the function of lncRNAs in the development of CRC (123). They identified a new lncRNA, called ADAMTS9-AS1, which was downregulated in CRC tissues, while the data retrieved from the TCGA demonstrated that it was significantly associated with clinical outcomes. Moreover, ADAMTS9-AS1 suppressed the proliferation of SW1116 and HT29 cells, induced cell cycle arrest, and inhibited tumor growth *in vivo*. Accordingly, the *in-silico* analysis suggested that lncRNA-ADAMTS9-AS1 upregulation preferentially targeted genes associated with cell proliferation and migration. Exploring the involved mechanism of action, the authors found that ADAMTS9-AS1 downregulated β-catenin, indicating that the Wnt pathway was involved in the regulatory role of ADAMTS9-AS1 on gene expression to suppress CRC tumorigenesis. Exosomal ADAMTS9-AS1 was shown to have diagnostic potential in CRC with an area under the curve (AUC) of 0.835 and a 95% confidence interval of 0.777–0.911 in the receiver operating characteristic (ROC) curve. Collectively, this study suggested that ADAMTS9-AS1 could suppress tumorigenesis in CRC through negative regulation of the Wnt signaling pathway. Accordingly, targeting this lncRNA could have prognostic and therapeutic potential in CRC patients, while exosomal ADAMTS9-AS1 could be a novel diagnostic biomarker (123).

Numerous human cancers have been shown to express the lncRNA H19 in large quantities. Compared with normal fibroblasts (NFs), cancer-associated fibroblasts (CAFs) in CRC have higher levels of H19 expression. Researchers discovered that the exosomes produced by CAFs might transfer the lncRNA H19 to nearby cells and activate the Wnt/β-catenin signaling pathway in CRC cells, to promote carcinogenesis and cell growth (107). The expression of lncRNA-UCA1 was higher in CRC patient tissues and plasma exosomes. Mechanistically, UCA1 can act as a ceRNA by sponging miRNA-143, thereby controlling the expression of MYO6. Researchers found that after treatment of CRC patient-derived cells with exosomal UCA1, the miR-143 expression was decreased, while the MYO6 expression was increased, thus promoting the growth of CRC cells (120). Exosomes may encapsulate lnc HEIH, which can subsequently be delivered into normal GI cells to induce the production of EZH2 and promote carcinogenesis by increasing the methylation of the GSDME promoter (81). Together, these findings suggest that dysregulation of exosomal lncRNAs might serve as biomarkers for CRC patients.

Exosomal lncRNA UCA1 controls cell proliferation in addition to mediating chemoresistance in CRC. Cetuximab-resistant cells expressed considerably more UCA1 than cetuximab-sensitive cells. Further research revealed that exosomal transfer of UCA1 from cetuximab-resistant CRC cells could increase the cetuximab resistance of recipient cells (195). lncRNA CCAL can increase the oxaliplatin (Oxa) and 5-FU resistance of CRC cells, and CAF-derived exosomes could deliver CCAL to these cells (196).

The clinical potential of the lncRNA RPPH1 in CRC patients has been investigated in the study of Liang et al. (121). Their findings revealed that RPPH1 was upregulated in CRC tissues and was correlated with advanced TNM stage and poor survival in CRC patients. The experimental analysis demonstrated that RPPH1 promoted tumor metastasis in cell and xenograft animal studies. Mechanistically, RPPH1 induced the EMT of CRC cells *via* interacting with and preventing β-III tubulin (TUBB3) ubiquitination. According to studies, overexpression of TUBB3 was functionally linked to enhanced cell motility and invasion of EMT-induced cells (197, 198). Moreover, exosomal RPPH1 derived from CRC cells was found to be transported into macrophages to mediate M2 polarization in recipient cells and promoted the proliferation and metastasis of CRC cells. Additionally, detectable plasma levels of exosomal RPPH1 were higher in treatment-naive patients, while they were reduced after tumor resection. Interestingly, plasma exosomal RPPH1 showed better diagnostic power (AUC = 0.86) in CRC patients compared with the conventional tumor markers CEA and CA-19-9. Taken together, this study suggested that RPPH1 could be a novel biomarker with diagnostic and therapeutic potential in CRC patients (121).

MELK belongs to the AMP-activated protein kinase-related kinase (AMPK) family, which is known to regulate various biological processes, such as cell cycle, proliferation, apoptosis, and cancer development (199–201). In CRC, MELK expression was found to be significantly higher in tumor specimens compared with healthy tissue and may contribute to cell cycle progression and cancer development (202). Exosomal lncRNAs have been reported to directly or indirectly control MELK expression levels in CRC (203). Zhao et al. reported that serum exosomal LINC02418 could be used as a potential diagnostic biomarker for CRC patients (118). They found that LINC02418 was upregulated both in cell lines and CRC tissues. Mechanistically, LINC02418 upregulated MELK *via* sponging miR-1273g-3p and acted as a ceRNA. Additionally, the diagnostic performance of cell-free LINC02418 and exosomal LINC02418 according to ROC and AUC curves revealed that exosomal LINC02418 could distinguish CRC patients from healthy individuals (AUC = 0.8978, 95% confidence interval = 0.8644–0.9351) with better performance compared with cell-free LINC02418 (AUC = 0.6784, 95% confidence interval = 0.6116–0.7452). Taken together, these findings demonstrated that LINC02418 was upregulated in CRC and played a role in CRC tumorigenesis *via* the miR-1273g-3p/MELK axis (118).

Natural killer (NK) cells are a substantial component of the immune system, which act as a surveillance system against tumors by eradicating cancer cells by direct killing or indirectly by secreting cytokines following their activation (204). NK cells can also inhibit tumor growth and metastasis. Some exosomal non-coding RNAs modulate the activity of NK cells (205). For instance, hepatocellular carcinoma (HCC) cell-derived exosomal circUHRF1 was demonstrated to induce NK cell exhaustion and reduce tumor infiltration by NK cells (206). Recently, a study has explored the mechanism involved in the exosome-mediated immune escape of CRC cells from attack by NK cells by transferring lncRNA cargoes (114). To establish an EMT model of SW480 cells, transforming growth factor beta (TGF-β) was used, and then the effect of the EMT-derived exosomes (EMT-exo) on the activity of NK cells was investigated. RNA sequencing was employed to identify the exosomal lncRNAs and target genes. The effect of exosomal lncRNAs on the proliferation of CRC tumors was confirmed *in vivo*. The results demonstrated that EMT-exo inhibited several characteristic functions of NK cells, including cell growth, cytotoxic activity, and IFN-γ production, as well as perforin-1 and granzyme B release. RNA sequencing also demonstrated the upregulation of lncRNA SNHG10 in EMT-exo relative to non-EMT-exo. Furthermore, SNHG10 was also upregulated in tumor tissues and was correlated with poor survival of CRC patients. Exosomal SNHG10 overexpression (oe-lnc-SNHG10 exo) inhibited NK cell viability and cytotoxicity. NK cell RNA sequencing showed upregulation of 114 genes in the oe-lnc-SNHG10 exo group, including the inhibin subunit beta C (INHBC), which plays a role in TGF-β signaling. INHBC knockdown reversed the effect of oe-lnc-SNHG10 exo on NK cells. oe-lnc-SNHG10 exo increased tumor growth through upregulation of INHBC *in vivo*, while it reduced the expression of perforin, granzyme B, and NK1.1 in cancer tissues. Collectively, this study showed that CRC cell-derived exosomal SNHG10 inhibited the antitumor activity of NK cells through upregulation of INHBC, suggesting that exosomal lncRNAs can help immune escape by suppressing NK cells and could be a potential therapeutic strategy for CRC (114).

The programmed death receptor 1 (PD-1) has been reported in several studies to mediate resistance to immune attack in cancer (207–209). The programmed death ligand 1 (PD-L1)/ubiquitin-specific protease 22 (USP22) axis has been found to control tumor immune escape (210–212). USP22 is considered to be an oncogene and can enhance the stability of substrate proteins through the inhibition of proteasomal degradation (213). PD-L1 was stabilized due to USP22 interactions. USP22 prevented PD- L1 proteasome degradation and led to its deubiquitination (211). It was discovered that exosomal lncRNA inhibited USP22 to facilitate PD-L1 ubiquitination (113). In this regard, the potential mechanistic involvement of lncRNA KCNQ1OT1 in tumor immune escape has been explored (113). Differentially expressed lncRNA and miRNAs were identified in healthy and tumor tissue samples using the GEO database and microarray analysis. RT-qPCR was employed to measure KCNQ1OT1, miR-30a-5p, USP22 as an oncogene, and PD-L1 expression levels. The interaction between KCNQ1OT1 and miR-30a-5p was confirmed by a dual-luciferase reporter assay and ribonucleoprotein immunoprecipitation (RIP) assay. Cell Counting Kit (CCK)-8, colony formation, scratch wound healing, and apoptosis assay were employed to assess tumor cell functions following treatment. The effect of USP22 on PD-L1 ubiquitination was studied by protein half-life assay and ubiquitination measurement. Finally, the effects of treatment on tumor growth and immune escape were explored in BALB/c mice and BALB/c nude mice. The results showed that KCNQ1OT1 was upregulated in tumor tissues and cancer cell-derived exosomes. KCNQ1OT1 overexpression promoted the malignant behavior of cancer cells due to the autocrine effect of cancer cell-derived exosomes. In turn, these exosomes mediated their effects through the miR-30a-5p/USP22 axis to modulate PD-L1 ubiquitination and suppress CD8^+^ T-cell responses, which eventually contributed to CRC development. Overall, this study found that KCNQ1OT1 was transferred by cancer cell-derived exosomes to regulate PD-L1 ubiquitination through the miR-30a-5p/USP22 axis to enhance CRC immune escape (113).

### Exosomal long non-coding RNAs and esophageal cancer

Esophageal squamous cell carcinoma (ESCC) is among the most aggressive malignancies, with a 5-year survival rate of only 15%–25% (214). ESCC patients often are diagnosed only at advanced stages and are resistant to conventional therapeutic regimens (50). Concurrent chemoradiotherapy (CCRT) is considered to be the standard therapy for these patients (215). A combination of cisplatin-based chemotherapy and radiotherapy (RT) has been shown in several randomized clinical trials to improve the 5-year survival rate in ESCC patients compared with those treated with RT alone (216). Although overall survival has been improved in patients receiving CCRT, cisplatin resistance often develops in patients with locally advanced cancer and is the main problem in the treatment of these patients (217). Exosomal lncRNAs have been reported in a number of studies to directly or indirectly participate in ESCC development and progression (133, 135, 218).

Fascin1 (FSCN1) is a member of the Fascin family and is involved in various biological processes like cell migration, motility, adhesion, and intercellular interaction (219). FSCN1 is differentially expressed in cancer cells, where it organizes actin filaments into pseudopodia and enhances ECM degradation by matrix metalloprotease (MMPs) and, therefore, contributes to the malignant phenotype and development and progression of tumors (220). The regulatory relationship between exosomal lncRNAs and cancer cell malignancy by regulating FSCN1 has been demonstrated (139). In this study, they investigated the potential effect of exosomal lncRNA LINC01711 on the treatment and prognosis of ESCC. Their results demonstrated that LINC01711 was upregulated in ESCC tissues, while its expression levels were associated with poor survival in patients (139). LINC01711 knockdown was shown to suppress the malignant phenotype of ESCC cells, while it also induced apoptosis. Mechanistically, LINC01711 sponged miR-326 to upregulate its target gene *fascin actin-bundling protein 1* (*FSCN1*). Moreover, xenograft animal studies demonstrated that exosomal LINC01711 (LINC01711-Exo) promoted tumor growth *in vivo*. Overall, exosomal LINC01711 enhanced the proliferation, migration, and invasion of ESCC cells through the miR-326/FSCN1 axis and, therefore, promoted ESCC development and progression (139).

The uncontrolled proliferation of cancer cells is the first step in tumor development. Exosomal lncRNAs have been discovered to facilitate this process in the tumor microenvironment (21). For instance, lncRNA ZFAS1 was higher in cancer tissues and could be transmitted between cancer cells *via* exosomes in ESCC. ZFAS1 was knocked down to explore its potential role in the malignant phenotype of ESCC cells. The expression of exosomal lncRNA ZFAS1 and the underlying mechanism involved in ESCC progression has been examined (133). A stable donor and recipient cell culture model was established. ZFAS1 silencing and overexpression, in addition to miR-124 inhibition experiments, were conducted to study their effects on cell proliferation, migration, and invasion and exosome trafficking in Eca109 cells. The *in- vitro* findings were confirmed by *in- vivo* experiments. The results demonstrated that ZFAS1 was upregulated and miR-124 was downregulated in ESCC tissues. ZFAS1 knockdown inhibited the proliferation, migration, invasion, while it increased the apoptosis in ESCC cells. Mechanistically, ZFAS1 acted by sponging miR-124 to upregulate STAT3. Exosomal ZFAS1 also was found to promote the malignant phenotype in ESCC cells, while it suppressed apoptosis and caused the same changes in STAT3 and miR-124 expression levels as seen for ZFAS1 overexpression. Additionally, exosomal ZFAS1 showed the same effect on tumor growth *in vivo*. Taken together, this study demonstrated that exosomal ZFAS1 promoted the malignant phenotype of ESCC cells through the miR-124/STAT3 axis and, thus, could contribute to ESCC development (133).

The tumor microenvironment (TME) has been found to play a substantial role in the development and progression of ESCC, similar to other cancers. In ESCC patients, the presence of fibroblast activation protein (FAP)-positive CAFs in the tumor stroma was reported to correlate with lymph node metastasis and poor prognosis (221). Studies have shown that adjacent NFs promote the recruitment and activation of CAFs (222, 223). However, CAFs have a greater potential to promote proliferation, invasion, and chemoresistance in various cancers compared with NFs (107, 224). Additionally, a number of studies have reported that CAFs can confer chemoresistance in cancer cells by releasing soluble factors such as interleukin-6 (IL-6), IL-11, hepatocyte growth factor (HGF), and TGF-β (225–228). The role of ESCC-derived exosomal lncRNAs in fibroblast activation, cancer progression, and cisplatin resistance has been shown (135). The results demonstrated that cancer cell-derived exosomal lncRNAs may contribute to cisplatin resistance in ESCC by transforming NFs into CAFs. Tumor tissues and normal esophageal epithelial tissues isolated from ESCC patients were used for the isolation of primary CAFs and matched NFs. Tong et al. investigated exosomal lncRNA transport between ESCC cells and NFs using fluorescence microscopy and qRT-PCR. To identify the lncRNAs involved in the process, the expression of 14 ESCC-related transcripts was analyzed in NFs following treatment with ESCC cell-derived exosomes. Their results revealed the transport of lncRNA POU3F3 from ESCC cells to NFs *via* exosomes and that exosomal POU3F3 contributed to fibroblast activation. In turn, activated fibroblasts enhanced the growth and cisplatin resistance of ESCC cells by secretion of IL-6. Furthermore, evaluation of POU3F3 expression in ESCC patients demonstrated a significant association between exosomal POU3F3 expression levels in plasma and the lack of complete responses and poor prognosis in ESCC patients. Overall, the findings in this study showed that exosomal POU3F3 promoted cisplatin resistance in ESCC, mediated *via* the transportation of exosomal POU3F3 from NFs to CAFs contributing to their transformation (135).

**Figure 5:**
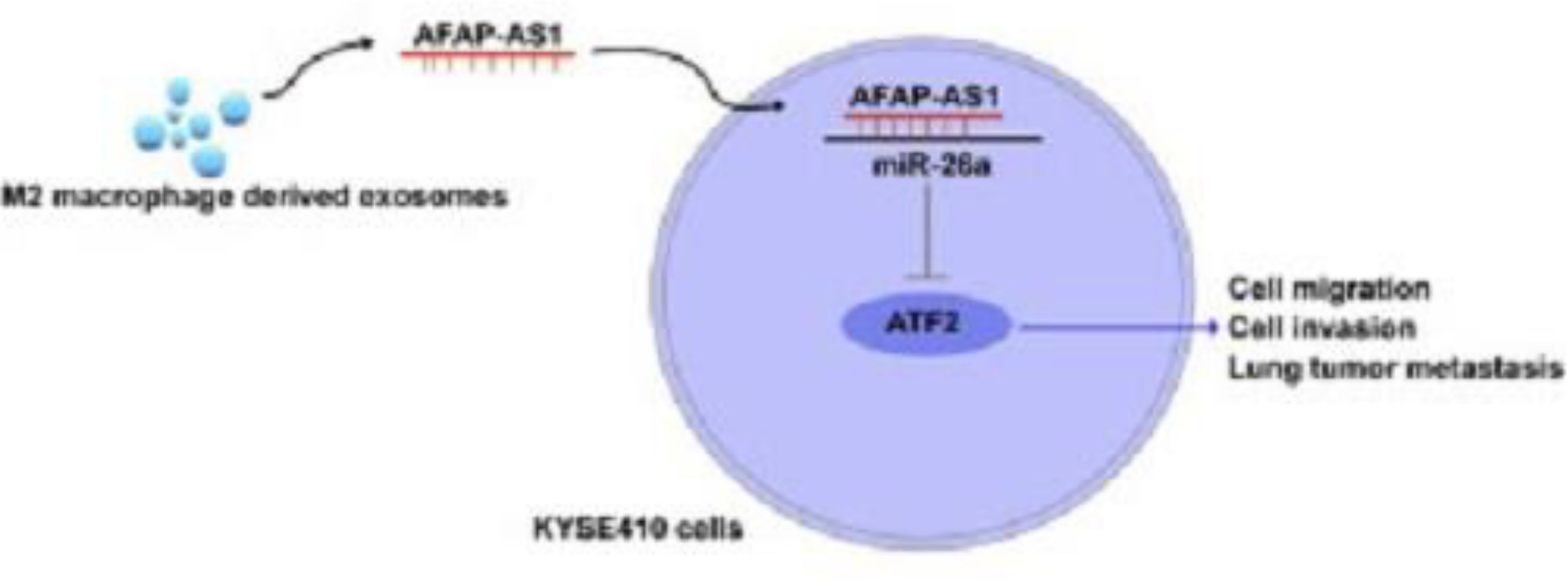
The M2 macrophage-derived exosomal lncRNA AFAP1-AS1 enhances the migration and invasion of EC cells and tumor metastasis through the AFAP1-AS1/miR-26a/ATF2 axis. Exosomes from immune or cancer cells can transport bio-macromolecules or lncRNAs to affect the development or progression of tumors by altering the microenvironment. ATF2 was a direct target of miR-26a, and lncRNA AFAP1-AS1 could selectively sponge miR-26a to reduce its expression. Exosomes produced by M2 macrophages had reduced miR-26a expression and greater AFAP1-AS1 and ATF2 expression. Additionally, extracellular AFAP1-AS1 might be transferred to KYSE410 cells by combining with exosomes produced by M2 macrophages. Through the high expression of AFAP1-AS1, M2 macrophage-derived exosomes may downregulate miR-26a and enhance the expression of ATF2, allowing EC cells to migrate, invade, and metastasize; M2-exosomes that increased AFAP1-AS1 or decreased miR-26a attenuated this effect. This figure was adapted from Mi et al. (137).

In another study, researchers investigated the effect of M2 macrophage-derived exosomes on the invasion and metastasis of esophageal cancer (EC) cells *via* the AFAP1-AS1/miR-26a/ATF2 axis (137). Their results showed that lncRNA AFAP1-AS1 targeted miRNA miR-26a to upregulate activating transcription factor 2 (ATF2). Moreover, they showed that AFAP1-AS1 and ATF2 were both upregulated, while miR-26a was downregulated in M2 macrophage-derived exosomes. These exosomes were able to transfer AFAP1-AS1 into EC cells in order to sponge miR-26a and then upregulate ATF2, to enhance the invasion and metastasis of EC (**Figure 5**). Taken together, they concluded that targeting M2 macrophages and the AFAP1-AS1/miR-26a/ATF2 axis may be a novel strategy for the treatment of EC (137).

### Exosomal long non-coding RNAs and oral cancer

Oral cancer arises in the lips or oral cavity. Most oral tumors originate from the squamous cells lining the organs called oral squamous cell carcinoma (OSCC) (229). Oral cancer tissues histologically can have various levels of differentiation and often show a tendency for metastasis to lymph nodes (230). Globally, oral and pharyngeal malignancies collectively are the sixth most commonly diagnosed types of cancer (231). Similar to other types of GI cancer, exosomal lncRNAs have been found to play a role in the development and progression of oral cancer (232).

lncRNAs can act either as tumor-suppressor genes or as tumor promoters or oncogenes. However, the development and spread of cancer is a multistep process that entails mutual autocrine–paracrine communications (e.g., controlled *via* secreted proteins or exosomes) between tumor cells and the nearby stromal cells (233, 234). CAFs are the most significant infiltrating non-immune cells within the stroma and promote tumor growth and metastasis in a paracrine manner (235). Other cell types such as bone marrow-derived mesenchymal stem cells, tissue adipocytes, circulating fibrocytes, and endothelial cells can also develop fibroblast markers during cancer progression and other inflammatory environments. However, CAFs are primarily produced from and activated by NFs. The roles of lncRNAs in CAFs are still not fully understood. The TME is a significant source of CAFs and can reprogram NFs so they can become protumorigenic CAFs (236–241). Therefore, it is necessary to understand how the stromal microenvironment affects CAF transformation. In this context, the exosomal lncRNA signature in stromal fibroblasts (NFs/CAFs) in OSCC was explored, including how the lncRNA profile changes during the NF/CAF transition (146). Ding et al. measured lncRNA expression levels in stromal fibroblasts and studied how they interacted with target miRNAs (146). RNA sequencing revealed a specific stromal lncRNA signature during the transformation of NFs into CAFs in OSCC tissues. Among them, a previously uncharacterized lncRNA was identified called FLJ22447, also known as LncRNA-CAF, which was significantly upregulated in CAFs. Interleukin-33 (IL-33) was mainly detected in the stromal space, where it showed a positive co-expression with Lnc-CAF to upregulate α-SMA, vimentin, and N-cadherin, which are CAF markers in fibroblasts. IL-33 silencing inhibited Lnc-CAF-mediated activation of stromal fibroblast and contributed to reduced proliferation of cancer cells. Mechanistically, Lnc-CAF increased IL-33 expression through inhibition of the p62-dependent autophagy–lysosome pathway without involving an lncRNA–protein complex. Autophagy induction using rapamycin suppressed the enhancing effect of Lnc-CAF/IL-33 on cell proliferation by promoting IL-33 degradation. Subsequently, the cancer cells further upregulated Lnc-CAF expression in stromal fibroblasts *via* exosomal transport. Clinical data demonstrated that high levels of Lnc-CAF/IL-33 in OSCC patients were correlated with advanced TNM stage and poor survival. Moreover, Lnc-CAF silencing suppressed tumor growth *in vivo* as shown by reduced Ki-67 expression and fewer stromal α-SMA- positive CAFs. Taken together, Ding et al. reported a stromal lncRNA signature with a role in the promotion of OSCC progression by transforming NFs into CAFs *via* exosomal Lnc-CAF/IL-33 transport (146).

### Exosomal long non-coding RNAs and hepatocellular carcinoma

Liver cancer accounts for the seventh most common human malignancy and the second deadliest. HCC is the most commonly diagnosed type of liver cancer, with a decreasing incidence in Asia and Italy, but an increasing pattern in the United States, European countries, and India (242). Chronic viral infections including hepatitis B virus (HBV) and hepatitis C virus (HCV) (243), along with non-infectious causes such as alcoholism, non-alcoholic fatty liver disease (NAFLD), and aflatoxin B1 consumption, are the main risk factors for HCC (244). Mechanistically, underlying factors have been identified to cause damage to the DNA, epigenetic changes, and development of some mutations related to the inactivation of tumor suppressor genes like *tumor protein p53* (*TP53*), *cadherin 1* (*CDH1*), and the *Ras association domain family member 1* (*RASSF1*). Moreover, activation of proto-oncogenes such as *MYC*, *VEGFA*, and *MAPK7* can lead to HCC progression (245–248). A number of studies have reported the role of exosomal lncRNAs in the development and progression of HCC (150, 158, 167, 249).

DLX6 antisense RNA 1 (DLX6-AS1) is an oncogenic lncRNA with a known function in the development and progression of various human malignancies like HCC (250). Wang et al. investigated the involvement and underlying molecular mechanism of exosomal DLX6-AS1 in HCC (160). The expression levels of DLX6-AS1, miR-15a-5p, and CXCL17 were measured in HCC cells and tissues. DLX6-AS1-overexpressing exosomes were isolated from HCC tissues and then co-cultured with M2 macrophages. MiR-15a-5p and CXCL17 silencing studies were conducted in macrophages. M2 macrophages were then co-cultured with HCC cells to assess the effect of exosomal DLX6-AS1-mediated macrophage polarization on the malignant phenotype of cancer cells *in vitro* and tumor metastasis *in vivo*. Next, DLX6-AS1/miR-15a-5p and miR-15a-5p/CXCL17 interactions were explored. The results demonstrated the upregulation of DLX6-AS1 and CXCL17, while miR-15a-5p was downregulated in HCC cells. HCC-exo positively regulated M2 macrophage polarization to promote migration, invasion, and EMT in HCC cells. These effects were enhanced by overexpression of DLX6-AS1; accordingly, DLX6-AS1 knockdown reversed these cellular properties. Furthermore, miR-15a-5p targeting enhanced M2 macrophage polarization and further promoted the invasion and metastasis of HCC. However, inhibition of the C-X-C motif chemokine 17 (CXCL-17) showed the opposite effects. It is known that in HCC, CXCL17 correlates with poor prognosis and low immune infiltration (251). CXCL17 is highly expressed in HCC tissues, and its overexpression promoted the migration and invasion of HCC cells, while its knockdown reversed these effects (252). Mechanistically, DLX6-AS1 was found to sponge miR-15a-5p and to upregulate CXCL17. Moreover, *in-vivo* results revealed that HCC cell-derived exosomal DLX6-AS1 enhanced lung metastasis through induction of M2 macrophage polarization. Overall, HCC cell-derived exosomal DLX6-AS1 regulated CXCL17 by sponging miR-15a-5p and, consequently, induced M2 macrophage polarization, which eventually promoted the migration, invasion, and EMT of HCC (160).

The Yes-associated protein 1 (YAP1) is a major protein functioning in the Hippo signaling pathway and regulates several biological processes like tissue regeneration, morphological characteristics, metabolism, and carcinogenesis, in addition to affecting intercellular communication, cell cycle surveillance, cell signaling, and cytoskeletal remodeling (253–257). Despite the fact that YAP1 has no direct DNA-binding activity, it is a known transcriptional co-activator, which regulates gene expression by interacting with other transcription factors. YAP1 can show dual functions in tumorigenesis, either as an oncogene contributing to tumor development or as a tumor- suppressor gene suppressing carcinogenesis. These contradictory effects depend on the precise transcription factor to which YAP1 binds and also on its subcellular localization (258). YAP1 has been reported to be highly expressed in blood vessels in HCC tissues, which suggests that YAP1 may play a role in angiogenesis (259). Nevertheless, the molecular mechanism by which YAP1 affects angiogenesis is not known and requires further investigation. YAP1 has been reported to be involved in regulating the expression of lncRNAs (260). Recently, researchers explored the potential role of YAP1 in exosomal lncRNA-mediated angiogenesis in HCC (161). They showed that YAP1 knockdown suppressed the proliferation and tube formation in vascular endothelial cells, suggesting that it could be a target for anti-angiogenesis therapy. YAP1 knockdown or inhibition also enhanced the release of the lncRNA MALAT1-loaded exosomes into the TME. When these exosomes were co-cultured with HCC cells, their invasion and migration were increased, which was found to be due to the activation of the extracellular signal-regulated kinase 1/2 (ERK1/2) signaling pathway. This study shed light on the underlying mechanism and novel pathways involving YAP1 in the development of distant tumor metastasis in anti-angiogenesis regimens and suggested novel targets to improve the long-term effectiveness of anti-angiogenesis treatment (161).

In a study by Li et al. (150), the authors reported that exosomal lncRNA TUC339 derived from HCC cells could regulate the activation and polarization of macrophages (150). Their findings showed high levels of the lncRNA TUC339 within HCC exosomes which could be transferred to THP-1 macrophage cells. TUC339 silencing increased pro-inflammatory cytokines, elevated the expression of co-stimulatory molecules, and increased phagocytosis in THP-1 cells, while its overexpression showed the opposite effects, indicating the role of TUC339 in regulating macrophage polarization. Furthermore, increased expression levels of TUC339 were detected in M2 macrophages compared with the M1 phenotype, while TUC339 was downregulated during the M2-to-M1 repolarization and vice versa. Accordingly, TUC339 silencing in IL-4-treated macrophages reduced the expression of M2 markers, while TUC339 overexpression in IFN-γ + LPS-treated macrophages enhanced these markers. These findings suggest that lncRNA TUC339 plays an essential role in regulating M1/M2 polarization in macrophages. Microarray analysis revealed that a variety of biological processes like cytokine–cytokine receptor interactions, CXCR chemokine receptor binding, Toll-like receptor (TLR) signaling, FcγR-mediated phagocytosis, cytoskeleton arrangement, and cell growth were associated with TUC339 expression in macrophages. Overall, this study revealed undiscovered regulatory functions of lncRNA TUC339 loaded into HCC cell-derived exosomes on the macrophage phenotype, in addition to complex interactions between cancer cells and immune cells *via* exosomal lncRNAs (150).

CD90 (Thy-1) is a GPI-anchored protein of 25– 37 kDa molecular weight, which is expressed and incorporated into the cell membrane of various cells, such as T lymphocytes, neurons, endothelial cells, and fibroblasts. CD90 has been found to play a role in intercellular interactions, cell–matrix interactions, apoptosis, cell adhesion, migration, fibrosis, and tumorigenesis (261). CD90 expression has been found to be elevated in hepatic stem/progenitor cells (262) and was associated with the malignant and differentiated phenotype of HCC cells in tumors (263) and a poor survival rate in HCC patients (264–266). Additionally, CD90^+^ HepG2 cells have shown aberrant expression of pro- and anti-apoptotic genes compared with CD90 ^−^ HepG2 cells (267). Recently, the exosome-mediated interactions of CD90^+^ HCC cells with endothelial cells have been investigated (151). Isolation and characterization of exosomes were conducted for both liver CD90^+^ cells and HCC cell lines. Isolated exosomes were used for the treatment of endothelial cells, which also were transfected with a plasmid encoding lncRNA H19. Then, the endothelial phenotype in treated cells was assessed using molecular and functional studies. The results revealed that the CD90^+^ cancer cell-derived exosomes affected the phenotype of endothelial cells to promote angiogenesis and intercellular adhesion. However, these effects were not seen for parental HCC cell lines. lncRNA profiling showed high expression of H19 in CD90^+^ cells, which was released inside exosomes. H19 silencing and overexpression demonstrated that H19 was involved in regulating the phenotype of endothelial cells through exosomes. Overall, these findings suggested a novel mechanism of action mediated by exosomes by which CSC-like CD90^+^ cells can affect the surrounding TME by enhancing angiogenesis. Additionally, the lncRNA H19 could be a potential therapeutic target in HCC (151).

## Conclusion

Exosomal lncRNAs are extremely important in many cancer-related processes, such as cell growth, metastasis, angiogenesis, immunomodulation, and drug resistance. Exosomal lncRNAs carry primary biological information that can be extracted to help understand, diagnose, and treat GI cancers. Exosomal lncRNAs can serve as possible cancer biomarkers since their levels often correlate with the disease severity in cancer patients. Exosomal lncRNAs, an emerging class of cancer biomarkers, have advantages over conventional markers. Exosomal lncRNAs can be employed as therapy targets in addition to helping with GI cancer diagnosis and prognosis. Exosomal lncRNAs are involved in the development of GI cancer and drug resistance; therefore, drugs which block their activity may be helpful in the treatment of cancer. However, even in recent studies, the importance of exosomal lncRNAs in GI cancer is only poorly understood. Exosomal lncRNAs are predicted to become more significant in the clinical management of GI cancer as further preclinical investigation progresses.

## Author contributions

HM was involved in the conception, design, statistical analysis, and drafting of the manuscript. MR, GB, MM, NZ, RS, RSa, DM, SS, FM, and MRZ contributed to data collection and manuscript drafting. All authors approved the final version of the manuscript for submission.
